# Erosive Vulvovaginitis Associated With *Borrelia burgdorferi* Infection

**DOI:** 10.1177/2324709619842901

**Published:** 2019-05-01

**Authors:** Melissa C. Fesler, Marianne J. Middelveen, Jennie M. Burke, Raphael B. Stricker

**Affiliations:** 1 Union Square Medical Associates, San Francisco, CA, USA; 2 Atkins Veterinary Services, Calgary, Alberta, Canada; 3 Australian Biologics, Sydney, New South Wales, Australia

**Keywords:** vulvovaginitis, Lyme disease, *Borrelia burgdorferi*, spirochetes, lichen planus, lichen sclerosus

## Abstract

We describe a case of acute erosive vulvovaginitis accompanying *Borrelia burgdorferi* infection. The patient is a 57-year-old woman previously diagnosed with Lyme disease who presented with a painful erosive genital lesion. At the time of the outbreak, she was being treated with oral antibiotics, and she tested serologically positive for *B burgdorferi* and serologically negative for syphilis. Histological examination of biopsy tissue from the lesion was not characteristic of dermatopathological patterns typical of erosive vulvar conditions. Dieterle-stained biopsy sections revealed visible spirochetes throughout the stratum spinosum and stratum basale, and anti–*B burgdorferi* immunostaining was positive. Motile spirochetes were observed by darkfield microscopy and cultured in Barbour-Stoner-Kelly–complete medium inoculated with skin scrapings from the lesion. Cultured spirochetes were identified genetically as *B burgdorferi sensu stricto* by polymerase chain reaction, while polymerase chain reaction amplification of treponemal gene targets was negative. The condition resolved after treatment with additional systemic antibiotic therapy and topical antibiotics. In cases of genital ulceration that have no identifiable etiology, the possibility of *B burgdorferi* spirochetal infection should be considered.

## Introduction

Erosive vulvovaginal conditions include a spectrum of inflammatory, infectious, and neoplastic processes of nonspecific morphologies.^1-4^ The etiologies of many of these conditions, including the lichenoid vulvar diseases lichen planus (LP) and lichen sclerosus (LS), are multifactorial and have not been fully elucidated, and cases of erosive vulvitis or genital ulceration without an identifiable etiology can occur.^1-4^ The diagnosis and treatment of erosive vulvar conditions is complicated by overlap of clinical characteristics and lack of knowledge concerning pathology and etiology.^1-3^ *Borrelia burgdorferi* has been associated with genital ulceration,^5^ so hypothetically it could cause an erosive vulvovaginal condition. We describe a case of erosive vulvovaginitis associated with *B burgdorferi* infection.

## Case Description

**Figure 1.**
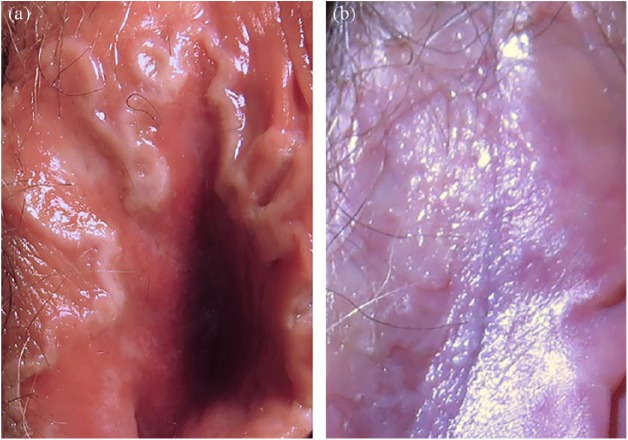
(a) Right labium majus and right labium minus showing a raw red ulceration with an elevated white epithelial margin. (b) Same view of right labium majus and right labium minus after antibiotic therapy.

The patient is a 57-year-old woman previously diagnosed with Lyme disease based on positive Lyme serological testing and systemic symptoms consistent with tickborne disease. While on treatment for Lyme disease with oral clarithromycin and cefdinir, she developed a painful erosive vulvovaginal ulceration consistent with conditions such as LP or LS. The ulceration encompassed the right labium minus, the right labium majus, the left labium minus, the vulvar vestibule, and the introitus (Figure 1a). The vulvar architecture was altered with partial loss and adhesion of the right labium minus. Routine culture for genital bacteria performed at a commercial laboratory was negative, and the patient was seronegative for syphilis. Therefore, further investigation to ascertain the cause of the condition was undertaken. The differential diagnosis included various lichenoid disorders, sexually transmitted infections, and hypersensitivities. After identification of *B burgdorferi* by culture and by histological examination, alternative antibiotic therapy was prescribed. The condition resolved after 5 months of treatment with topical clindamycin and oral doxycycline (Figure 1b).

## Materials and Methods

The detection of *Borrelia* spirochetes was accomplished by both culture and by direct examination of histological sections. A biopsy was taken from the margin of the erosion, fixed in formalin, and then blocked, sectioned, and stained at McClain Laboratories LLC (Smithtown, NY). Sections were stained with hematoxylin and eosin (H&E), Dieterle silver nitrate stain, and anti–*B burgdorferi* immunostain (Abcam), as previously described.^6,7^ Vaginal and skin cultures of *Borrelia* were performed as previously described by inoculation of vaginal secretions or vulvar skin scrapings from the ulceration into Barbour-Stoner-Kelly H–complete medium with 6% rabbit serum (Sigma Aldrich, #B8291) and the following antibiotics: phosphomycin (0.02 mg/mL; Sigma Aldrich), rifampicin (0.05 mg/mL; Sigma Aldrich), and amphotericin B (2.5 µg/mL; Sigma Aldrich).^6,7^ Polymerase chain reaction (PCR) amplification of *Borrelia* DNA and Sanger sequencing of the vaginal culture were performed at Australian Biologics Laboratory as described previously.^6,7^ Culture pellets were obtained by centrifugation, stabilized in AL buffer (Qiagen), and then forwarded to Australian Biologics for *B burgdorferi* detection and identification. DNA was extracted using the QIAamp DNA Mini Kit (Qiagen). *Borrelia* DNA was detected using real-time PCR targeted to the *B burgdorferi* 16S rRNA gene and endpoint PCR targeted to the rpoC gene.^6,7^

## Results

### Histological Findings

**Figures 2.**
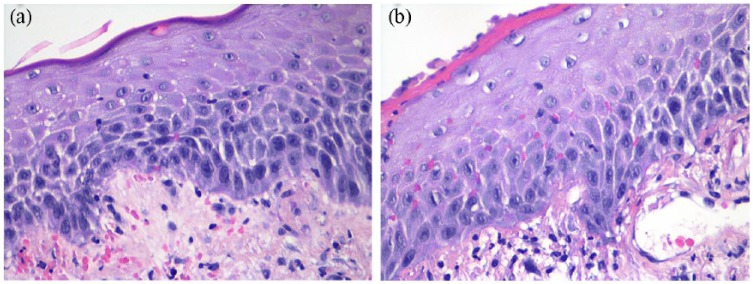
(a and b) H&E stain of epidermis and dermis demonstrating hemorrhage, a diffuse lymphocyte and PMN infiltrate, and enlarged nuclei and mitosis in the basal layer, at 200× and 400× magnification, respectively.

**Figures 3.**
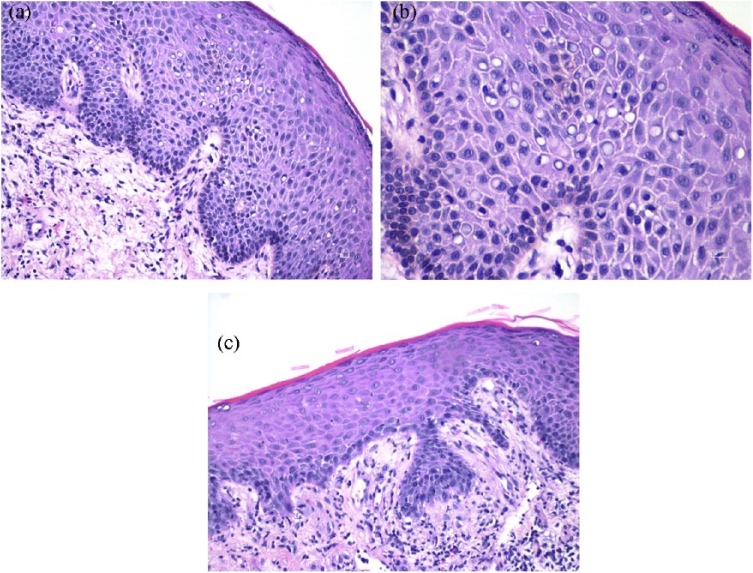
(a and b) Epidermis, demonstrating spongiosis, and perinuclear clearing, at 200× and 400× magnification, respectively. (c) Epidermis, demonstrating spongiosis, perinuclear clearing, and parakeratosis, at 100× magnification.

Histological examination revealed that the ulceration was not characteristic of a lichenoid reaction such as LP or LS, or any other standard dermatopathology reaction patterns. H&E staining demonstrated intraepidermal and dermal hemorrhage with a diffuse infiltrate of lymphocytes and neutrophils (Figures 2a and 2b). The stratum basale had a reactive appearance with some enlarged nuclei and mitoses, but did not have a degenerative appearance such as vacuolar change, apoptotic bodies, or squamatization. The epidermis showed spongiosis, perinuclear clearing (Figures 3a and 3b), and parakeratosis (Figure 3c). A reactive basal layer has been described in erosive LP,^8^ but, in contrast to our case, regenerative erosive LP features a thinned/eroded epithelium accompanied by a more concentrated band-like lymphocytic infiltrate and does not include hemorrhage as a prominent component.

**Figure 4.**
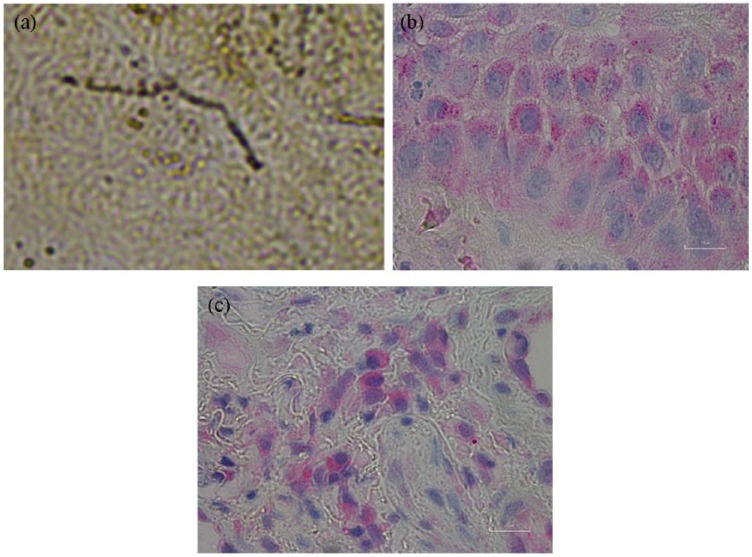
Dieterle staining and anti–*B burgdorferi* immunostaining at 1000× magnification. (a) Long *B burgdorferi* spirochetes in the stratum spinosum of the epidermis. (b) Intracellular anti–*B burgdorferi* immunostaining within basal keratinocytes of the epidermis. (c) Intracellular anti–*B burgdorferi* immunostaining within dermal macrophages.

There was little or no Dieterle staining in the stratum corneum or upper layers of the epidermis, indicating that infection was not superficial but was established in the deeper layers of the epidermis and dermis. Dieterle staining concentrated predominantly among the keratinocytes in the stratum basale, staining both long spirochetes and intracellular organisms characteristic of different morphological variants of *B burgdorferi*. Long spirochetal forms occurred within the stratum spinosum (Figure 4a), while scattered round variants consistent with cystic morphologic forms of *B burgdorferi* occurred within the dermis. Spirochetes were more frequent in areas with perinuclear clearing and were found surrounding necrotic vacuoles within the tissue.

Anti–*B burgdorferi* immunostaining was positive mainly within keratinocytes in the basal layer of the epidermis (Figure 4b). There was also some immunostaining in the dermis with mostly intracellular staining of macrophages (Figure 4c).

### Culture

**Figure 5.**
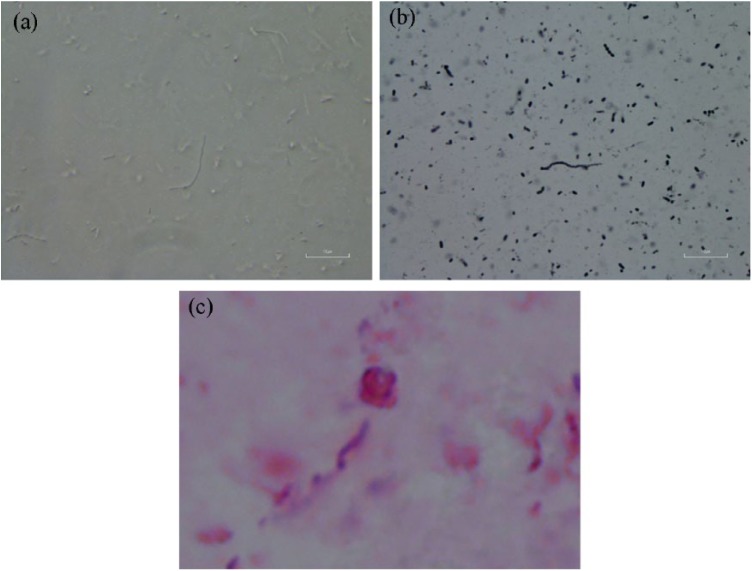
Vulvar skin culture containing visible spirochetes at 1000× magnification. (a) Viable spirochetes in culture inoculated with vulvar skin scrapings observed in culture fluid at 1 week using darkfield microscopy. (b) Dieterle stain showing spirochete morphologically consistent with *B burgdorferi* and spherical variants. (c) Anti-*B burgdorferi* immunostain reactive with cultured spirochetes.

Motile, viable spirochetes and sessile spherical bodies were observed in culture fluid inoculated with either vaginal secretions or genital skin scrapings when examined by darkfield microscopy at 1 week of incubation (Figure 5a). Spirochetes and spherical morphological forms characteristic of *B burgdorferi* were observed in Dieterle-stained smears (Figure 5b), and anti–*B burgdorferi* immunostain reacted positively to culture smears (Figure 5c). PCR amplification followed by Sanger sequencing genetically identified the cultured isolate as *B burgdorferi sensu stricto*. PCR amplification of *Treponema pallidum* and *Treponema denticola* gene targets was negative (data not shown).

## Discussion

We describe a case of an erosive vulvovaginal lesion where a known cause was lacking. The histological pattern was distinct from standard dermatopathological reactions including the histopathology associated with lichenoid reactions and herpetic lesions.^8,9^ *Borrelia* infection was identified based on immunohistological, culture, and molecular techniques, suggesting that the pathology may have been associated with spirochetal infection. The lesion occurred despite the ongoing use of antibiotic therapy, and it resolved with site-directed systemic and topical antibiotics. Persistent *Borrelia* infection despite antibiotic therapy has recently been described, and survival of the spirochete in privileged sites such as the genital tract has been postulated.^7,10^ *B burgdorferi* has been cultured from vaginal and seminal secretions of Lyme disease patients, and the fact that active infection was present in an erosive genital lesion suggests that sexual transmission of Lyme disease may be possible. Although previous studies in animal models suggest that sexual transmission of *B burgdorferi* may occur, further work is needed to examine this possibility.^11^

## Conclusions

In cases of genital ulceration that have no identifiable etiology, the possibility of *B burgdorferi* spirochetal infection should be considered. The association between erosive genital lesions and *Borrelia* infection merits further study.
